# Acute Bilateral Superior Branch Vestibular Neuropathy

**DOI:** 10.3389/fneur.2018.00353

**Published:** 2018-05-17

**Authors:** Dario A. Yacovino, John B. Finlay, Valentina N. Urbina Jaimes, Daniel H. Verdecchia, Michael C. Schubert

**Affiliations:** ^1^Department of Neurology, Cesar Milstein Hospital, Buenos Aires, Argentina; ^2^Memory and Balance Clinic, Buenos Aires, Argentina; ^3^Princeton University, Princeton, NJ, United States; ^4^Universidad Maimónides, Área de Rehabilitación Vestibular, Buenos Aires, Argentina; ^5^Departamento de Ciencias de la Salud, Kinesiología y Fisiatría, Universidad Nacional de La Matanza (UNLaM), Buenos Aires, Argentina; ^6^Johns Hopkins University, Otolaryngology, Baltimore, MD, United States; ^7^Johns Hopkins University, Physical Medicine and Rehabilitation, Baltimore, MD, United States

**Keywords:** vestibular neuritis, vestibulo-ocular reflex, head impulse test, bilateral vestibular hypofunction, acute gait ataxia

## Abstract

The rapid onset of a bilateral vestibular hypofunction (BVH) is often attributed to vestibular ototoxicity. However, without any prior exposure to ototoxins, the idiopathic form of BVH is most common. Although sequential bilateral vestibular neuritis (VN) is described as a cause of BVH, clinical evidence for simultaneous and acute onset bilateral VN is unknown. We describe a patient with an acute onset of severe gait ataxia and oscillopsia with features compatible with acute BVH putatively due to a bilateral VN, which we serially evaluated with clinical and laboratory vestibular function testing over the course of 1 year. Initially, bilateral superior and horizontal semicircular canals and bilateral utricles were impaired, consistent with damage to both superior branches of each vestibular nerve. Hearing was spared. Only modest results were obtained following 6 months of vestibular rehabilitation. At a 1-year follow-up, only the utricular function of one side recovered. This case is the first evidence supporting an acute presentation of bilateral VN as a cause for BVH, which would not have been observed without critical assessment of each of the 10 vestibular end organs.

## Introduction

Acute vestibular syndrome (AVS) is a clinical condition characterized by sudden, severe, and prolonged vertigo that develops over seconds, minutes, or hours. AVS of peripheral origin is a result of the asymmetric vestibular nerve input due to acute unilateral vestibular nerve or labyrinthine damage (1). Patients often have a presumed viral or immune related cause for their symptoms of AVS. Vestibular neuritis (VN) is the most accepted etiology when hearing is spared, with the lesion presumed to be localized to the vestibular nerve (vestibular neuropathy) (2). While rare, a separate lesion to the contralateral nerve has been described in 1–4% of patients (2, 3). Such cases usually occur in a sequential pattern, after a long period (months to years) following the initial nerve damage (2, 4). To our knowledge and experience, simultaneous bilateral and acute involvement of each vestibular nerve due to a putative VN is undocumented (5).

Acute bilateral vestibular damage is rare and has generally been associated with the iatrogenic effect of ototoxic drugs such as gentamicin (5). Typical symptoms include blurred vision induced by head movement (oscillopsia) and gait ataxia. In these cases, the ototoxicity of the hair cells is diffuse, causing a global loss of function easily identified using vestibular function tests (6). Here, we report a case of acute bilateral vestibular hypofunction (BVH) with selective damage to each superior vestibular nerve branch. We propose the mechanism is a bilateral simultaneous VN, unreported to date in the literature.

## Case Report

A 68-year-old man with a 7-day history of upper respiratory tract infection had no prior history of vertigo, gait, or hearing disorder. He began to suffer from vertigo that developed over minutes. The following day, he reported his vertigo resolved but required help with walking due to a severe ataxia. Furthermore, he reported that images of the visual environment appeared blurry during head motion. He did not suffer any subjective changes in hearing. The patient was seen in the emergency room where he had no limb ataxia or other motor or sensory abnormalities. A 1.5 T MRI (1.5 T Achieva; Philips, Eindhoven, Netherlands) with 3D FLAIR sequence of the brain stem and cerebellum revealed non-specific isolated cerebral white matter lesions. Diffusion weighted sequences (DW1) and T1 with contrast was normal. A more detailed neuro-otologic exam with Video Frenzel goggles was performed on the third day from onset. No bedside ocular-motor abnormalities were observed (no nystagmus, normal pursuit, and saccades). With fixation removed, the patient had a spontaneous upbeating nystagmus. The Dix–Hallpike test induced an increase in the intensity of the spontaneous nystagmus without positional vertigo. The clinical head impulse test (HIT) was abnormal bilaterally in the horizontal semicircular canal plane. The dynamic visual acuity test showed a loss from baseline of eight lines for horizontal head rotation. In the Romberg test, the patient fell backward with his eyes closed and during head motion. He was unable to walk without falling.

**Figure 1:**
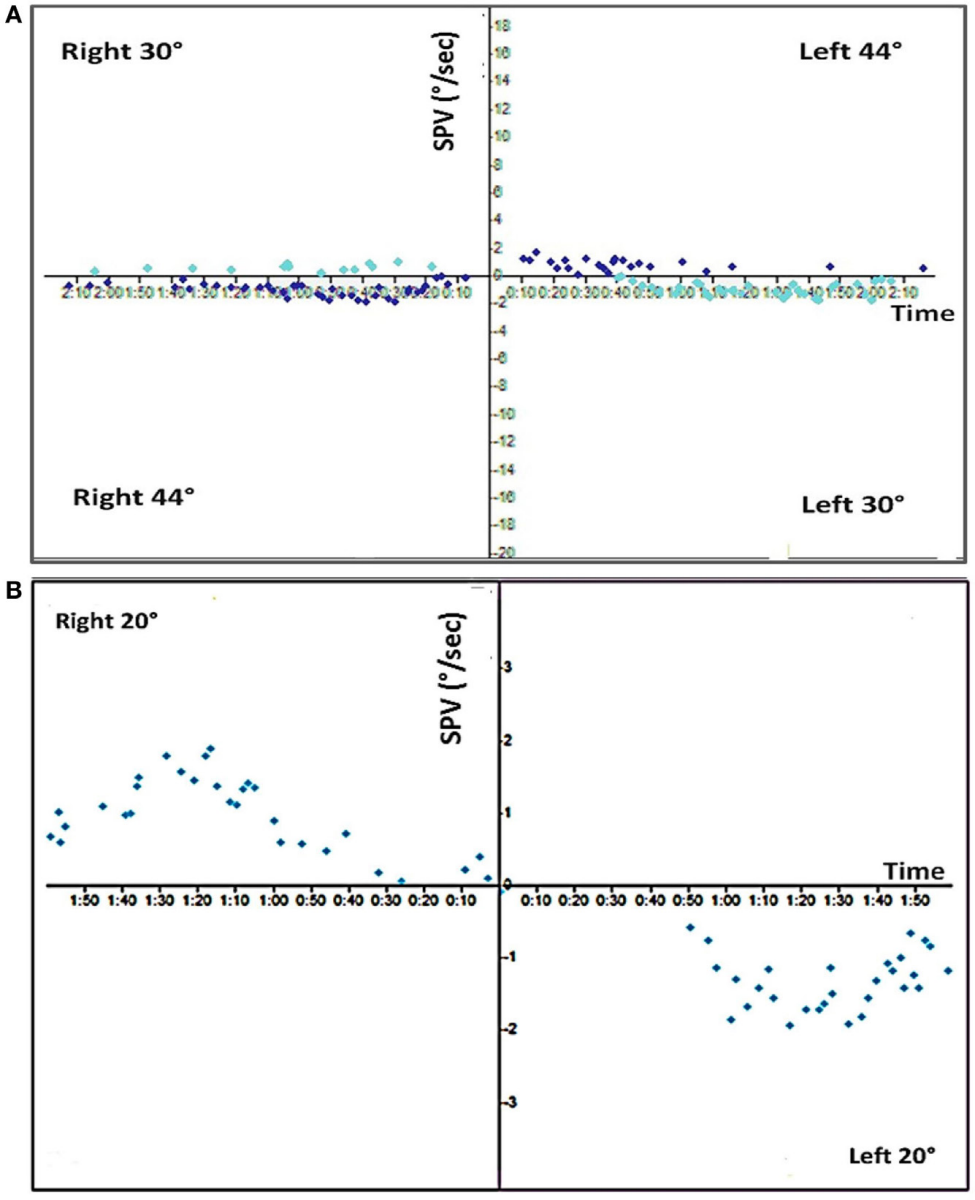
Caloric exam. **(A)** Top, the standard bithermal caloric test showed no response to either temperature irrigation from either ear. **(B)** Bottom, ice water irrigation (20°C) shows only a minor residual response (total response = 4°/s).

**Figure 2:**
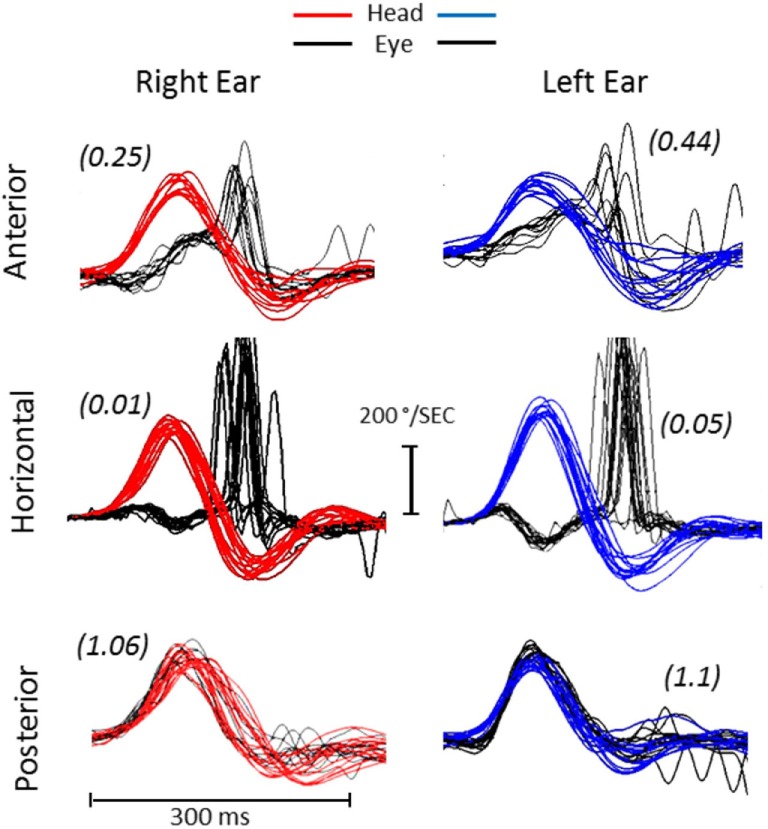
Video head impulse test (vHIT). The vHIT shows severe and reduced vestibulo-ocular reflex (VOR) gain in the horizontal and superior semicircular canals with corrective compensatory saccades. Both posterior semicircular canals (RP, right posterior; LP, left posterior) show normal VOR gain (parenthesis) without compensatory saccades.

Standard video-nystagmography (VO425-Interacoustics; Middelfart, Denmark) revealed normal smooth pursuit, saccades, and optokinetic nystagmus. However, bithermal caloric testing with water irrigation revealed a bilateral vestibular areflexia, with very weak, though residual responses to ice water irrigation (Figure 1). Video head impulse test (vHIT) (EyeSeeCam—Interacoustics, Middelfart, Denmark) showed a severe reduction in each horizontal and superior semicircular canal gains but a normal response in both posterior semicircular canal gains (Figure 2). The patient was started on a steroid taper over 2 weeks.

**Figure 3:**
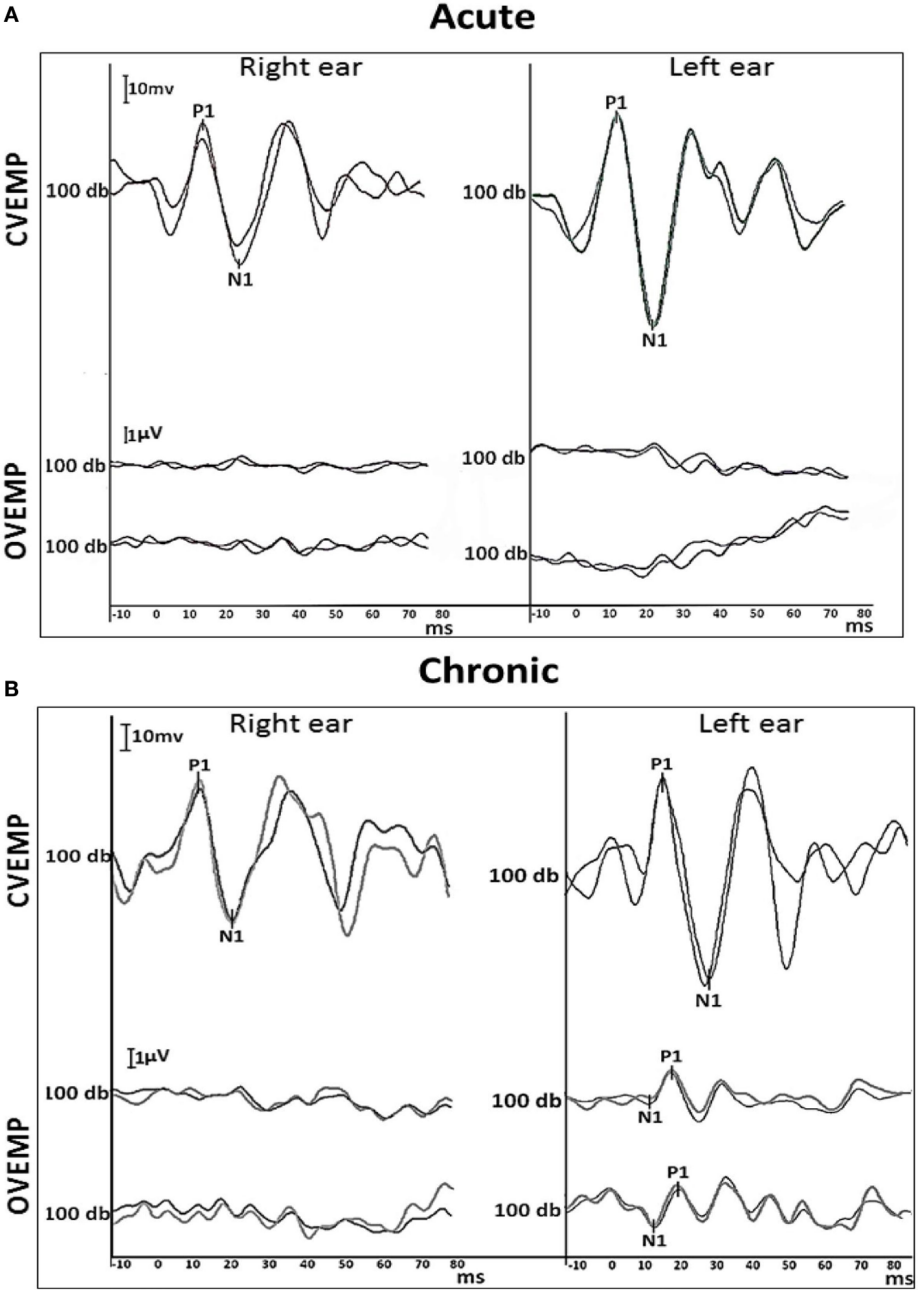
Air-conducted VEMP traces. **(A)** Acute cervical (top) and ocular (bottom) VEMP responses recorded 2 weeks from the onset of symptoms. **(B)** Chronic cervical (top) and ocular (bottom) VEMP recorded again at 12 months. The cVEMP responses were reproduced bilaterally at the acute and chronic stages with latency, amplitude and asymmetry within the normal range. The cVEMP amplitudes [normalized to background electromyography (EMG) activation (EMG scaling)], showed <20% asymmetry, suggesting bilaterally spared inferior vestibular nerves. In contrast, the acute oVEMP showed no reproducible responses bilaterally. At 1 year, a reproducible oVEMP was observed only on the left side. Two trials were conducted in order to confirm results (two traces). Cervical and ocular VEMP waves of respective potentials (positive and negative deflections—P1/N1) were analyzed. Stimuli was a 100 db air-conducted 500 Hz tone burst.

**Figure 4:**
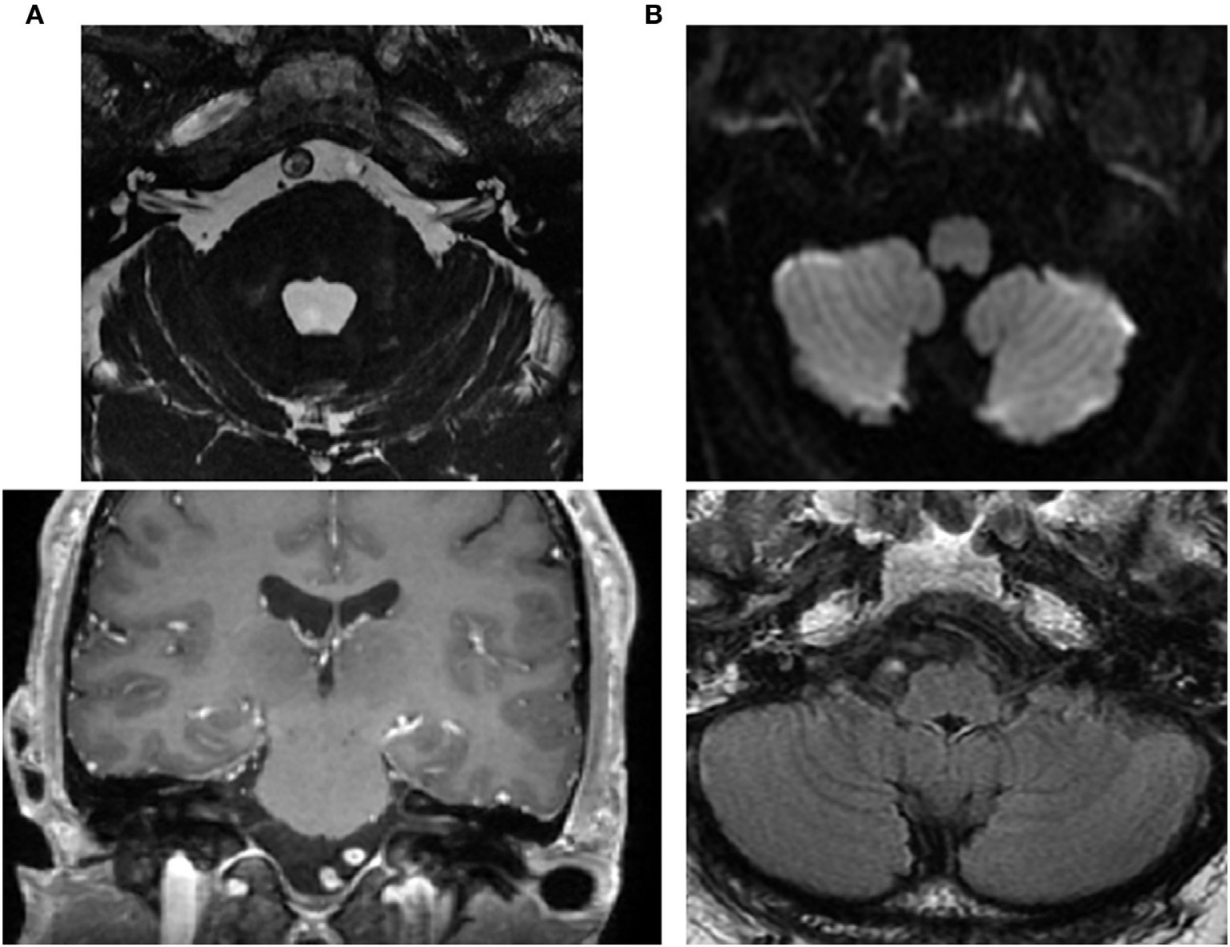
Brain MRI. **(A)** Normal internal auditory canals as visualized *via* axial FIESTA (upper) and coronal T1 with contrast (lower). **(B)** Normal inferior and medial vestibular nuclei at the level of the medulla (axial diffusion—upper) and FLAIR (lower).

In a follow-up consultation 2 weeks later, air-conducted cervical and ocular vestibular evoked myogenic potential (cVEMP and oVEMP) (Eclipse-Interacoustics, Middelfart, Denmark) showed an absence of both oVEMP responses but normal cVEMP responses (Figure 3). The audiogram showed a mild, symmetrical (left–right) high frequency hearing loss with normal auditory evoked potentials. Extensive serological studies testing for paraneoplastic syndromes, auto-immunological panel including anti-cochlear antibody, viral hepatitis (A, B, C), Epstein–Barr virus, herpes simplex virus 1 and 2, varicella-zoster virus, HIV, human cytomegalovirus, venereal disease research laboratory test, and mycoplasma pneumonia were all negative. A second MRI 3 tesla (3 T Discovery 750, General Electrics, Milwaukee, WI, USA) did not show any changes within the auditory canal or of the brain structures (Figure 4).

The patient was seen again 30 days from initial examination with report of a positional vertigo. The Dix–Hallpike maneuver confirmed a posterior semicircular canal benign paroxysmal positional vertigo (PC-BPPV) on the right side. A repositioning maneuver (Modified Epley) resolved the PC-BPPV.

The patient completed a 5-month vestibular rehabilitation program including home exercises focused on vestibulo-ocular reflex (VOR) and balance exercises with only modest results (Table 1). At 6 months, the spontaneous upbeating nystagmus without visual fixation had disappeared. However, a new left-sided PC-BPPV was diagnosed, treated, and resolved. At 1-year follow-up, the patient was symptom-free at rest and low-velocity walking but reported oscillopsia during running or high-velocity head movement. Repeat vHIT did not show any change compared to earlier studies (Table 1). An improvement in the left oVEMP was documented (Figure 3).

**Table 1:** Comparison between pre- and post-vestibular rehabilitation program.

Evaluation	Pre	Post
Dizziness handicap inventory (DHI)	
– Total	64	48
– Emotional	18	12
– Functional	22	18
– Physical	24	18
Activities-specific balance confidence scale (ABC)	85%	88.12%
Oscillopsia visual analog scale (oVAS) while walking (0–10)	8.20	7.50
Modified clinical test for sensory interaction in balance (CSTIB)	90/120 s	90/120 s
Clinical vestibular dynamic visual acuity (DVA)—4 m	
– Difference from static acuity yaw plane	8 lines	7 lines
– Difference from static acuity pitch plane	5 lines	4 lines
Gait speed (comfortable) m/s	1.10	1.14
Functional gait assessment (FGA)	22/30	23/30
Vestibulo-ocular reflex Gain (vHIT)	
– aSCC (right/left)	0.25/0.44	0.36/0.42
– hSCC (right/left)	0.01/0.05	0.03/0.04
– pSCC (right/left)	1.06/1.10	1.03/1.12

*Pre and 6-month post outcome measures of vestibular physical therapy. Modified CSTIB results reflect duration of time in standing on firm and foam surface with eyes open and closed (120 s is normal). VHIT gain results of all 6 canals did not change. aSCC, anterior semicircular canal; hSCC, horizontal semicircular canal; pSCC, posterior semicircular canal*.

Written informed consent was obtained from the participant for publication of this case report.

## Discussion

Bilateral vestibular hypofunction is characterized by unsteadiness of posture and gait with a disabling oscillopsia during head movements. Objective testing of the VOR using laboratory vestibular function tests confirms the BVH (7). Our case presents a clinical picture most compatible with acute BVH with preservation of hearing given the absence of a central nervous system lesion, hearing loss, or history of ototoxicity exposure. VN is typically unilateral and damages the superior vestibular nerve much more frequently than the inferior vestibular nerve (8). We propose our case is best explained as a bilateral VN, given that the pattern of damage has clearly damaged both superior vestibular nerves—as evidence from the reduction of VOR gain from each horizontal and superior semicircular canals and the utricle, with preservation of function in the posterior semicircular canal and saccule.

Substantial clinical features rule out a sequential VN as the first diagnostic: it is highly unlikely that the patient had a previous unilateral VN for three main reasons. First, there was no previous history of vertigo, dizziness, or any ancillary related symptoms like hearing disorders, gait unsteadiness, or BPPV as would be expected in the case of a prior unilateral reduction of vestibular function. Second, the symptoms of an acute unilateral vestibular hypofunction are accompanied with a mixed horizontal and torsional nystagmus that beats toward the more active labyrinth, which reduces once the asymmetrical vestibular tone is restored by central compensation. In a sequential VN, the second assault then causes the nystagmus to beat in the contralateral direction due to central decompensation (9) [known as Bechterew’s phenomenon (10)]. Although our patient did experience a brief vertigo, likely signifying a brief asymmetric involvement as both systems were suffering impairment at different rates, our patient instead had a spontaneous upbeating nystagmus. In a bilateral superior vestibular neuropathy, a severe reduction of input from the horizontal and superior semicircular canals along with the utricles creates a bias in the neural resting activity of the residual posterior semicircular canals and saccule. This pattern of symmetric and unopposed vertical semicircular canal excitation results in an upbeat spontaneous nystagmus without a torsional component due to the roll components canceling, as described in the case of bilateral posterior canal stimulation (11). Finally, our patient developed BPPV, a common complication (16%) of VN of the superior branch given the spared posterior semicircular canal (3). This is attributed to utricular damage that causes detached otolithic debris to fall into the neutrally active and ipsilateral posterior semicircular canal (12). Our patient had BPPV of both posterior semicircular canals during the follow-up, which provides additional evidence of damage to the superior branch without inferior branch involvement of the vestibular nerves.

Vestibular rehabilitation is known to help patients with BVH, though the range of meaningful change is highly variable and their quality of life often remains impaired (13, 14). Our patient did not have much improvement in the outcome measures we examined, matching prior report of stable function and highlighting the need for different forms of rehabilitation (15, 16).

## Conclusion

Our case report suggests that (1) VN can present bilaterally and suddenly; (2) in patients with acute onset of severe ataxia and oscillopsia, the clinician should rule out acute bilateral VN; (3) loss of caloric function by itself is insufficient to diagnose bilateral VN given its limit in identifying the spared vestibular function; and (4) therefore, examining each of the 10 vestibular end organs with VHIT and VEMPs in acute case presentations of BVH is the only way to identity of bilateral vestibular neuropathy as an independent condition.

## Ethics Statement

This study was carried out in accordance with the University of Maimónides ethical standards. All subjects gave written informed consent in accordance with the Declaration of Helsinki.

## Author Contributions

DY, VUJ, and DV oversaw the patient and collection of data. DY, JF, DV, and MS wrote and critically revised the manuscript.

## Conflict of Interest Statement

The authors declare that the research was conducted in the absence of any commercial or financial relationships that could be construed as a potential conflict of interest.
